# Sheldon-Hall syndrome

**DOI:** 10.1186/1750-1172-4-11

**Published:** 2009-03-23

**Authors:** Reha M Toydemir, Michael J Bamshad

**Affiliations:** 1 Howard Hughes Medical Institute, Department of Human Genetics, University of Utah, Salt Lake City, UT, USA; 2 Departments of Pediatrics and Genome Sciences, University of Washington, Seattle, WA, USA; 3 Seattle Children's Hospital, Seattle, WA, USA

## Abstract

Sheldon-Hall syndrome (SHS) is a rare multiple congenital contracture syndrome characterized by contractures of the distal joints of the limbs, triangular face, downslanting palpebral fissures, small mouth, and high arched palate. Epidemiological data for the prevalence of SHS are not available, but less than 100 cases have been reported in the literature. Other common clinical features of SHS include prominent nasolabial folds, high arched palate, attached earlobes, mild cervical webbing, short stature, severe camptodactyly, ulnar deviation, and vertical talus and/or talipes equinovarus. Typically, the contractures are most severe at birth and non-progressive. SHS is inherited in an autosomal dominant pattern but about half the cases are sporadic. Mutations in either *MYH3*, *TNNI2*, or *TNNT3 *have been found in about 50% of cases. These genes encode proteins of the contractile apparatus of fast twitch skeletal muscle fibers. The diagnosis of SHS is based on clinical criteria. Mutation analysis is useful to distinguish SHS from arthrogryposis syndromes with similar features (*e.g*. distal arthrogryposis 1 and Freeman-Sheldon syndrome). Prenatal diagnosis by ultrasonography is feasible at 18–24 weeks of gestation. If the family history is positive and the mutation is known in the family, prenatal molecular genetic diagnosis is possible. There is no specific therapy for SHS. However, patients benefit from early intervention with occupational and physical therapy, serial casting, and/or surgery. Life expectancy and cognitive abilities are normal.

## Disease name and synonyms

Sheldon-Hall syndrome (SHS);

Distal arthrogryposis type 2B (DA2B);

Freeman-Sheldon syndrome variant;

Arthrogryposis multiplex congenita, type II.

## Definition

Sheldon-Hall syndrome (SHS, MIM# 601680) or distal arthrogryposis type 2B (DA2B) is an autosomal dominant disorder characterized by congenital contractures of the distal joints of the limbs without a primary neurological defect. SHS is similar to another distal arthrogryposis called Freeman-Sheldon syndrome (FSS or DA2A), first described by Freeman and Sheldon in 1938, but distinguished as a separate entity in 1997 [1,2]. The most common clinical features of SHS include a triangular face, downslanting palpebral fissures, prominent nasolabial folds, small mouth, high arched palate, attached earlobes, mild cervical webbing, short stature, severe camptodactyly, ulnar deviation, and vertical talus and/or talipes equinovarus [2].

## Epidemiology

The prevalence of arthrogryposis is about 1/3000 [3]. Epidemiological data for the distal arthrogryposis (DA) syndromes are not available, but anecdotal data suggest that SHS is the most common of the DAs and the most frequent cause of heritable arthrogryposis. The prevalence of DA appears to be similar across different populations although most of the individuals reported to date are of European ancestry. There is no known sex bias.

## Clinical description

**Figure 1:**
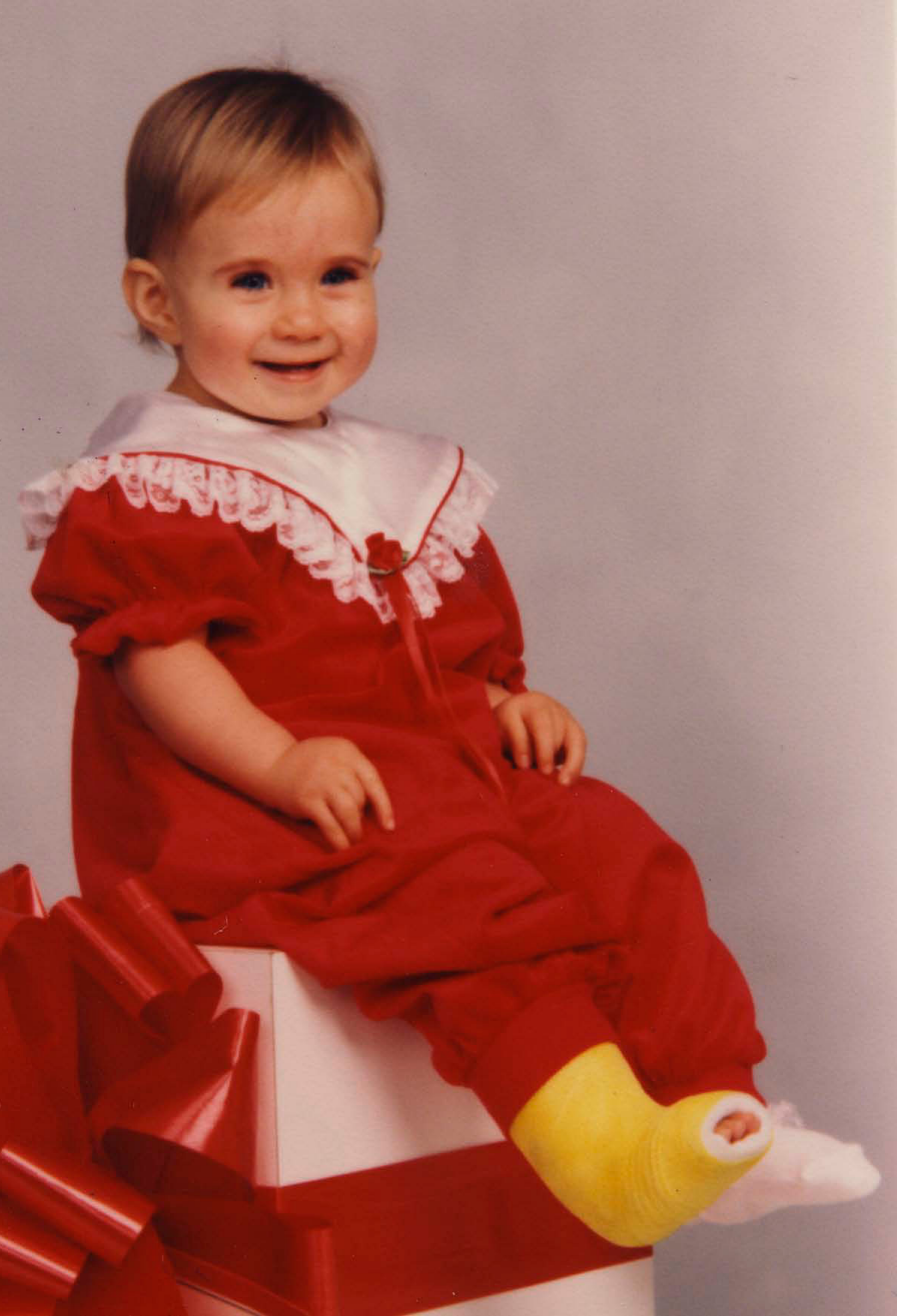
**Clinical features of Sheldon-Hall syndrome**. Note the pointed chin, long philtrum, prominent nasolabial folds, downslanted palpebral fissures, attached ear lobes. She also has camptodactyly and ulnar deviation at the wrist. The right foot was casted to treat talipes equinovarus.

The major clinical features of SHS are summarized in Table 1. The clinical presentation is highly variable both within and between families. Still, in most cases the clinical diagnosis of SHS is based on the pattern of contractures observed at birth, which are usually limited to hands and feet. Most patients have camptodactyly, ulnar deviation and/or overlapping of the fingers, a narrow and triangular face, and overlapping toes (Figure 1). The contractures are virtually always worse at birth and non-progressive.

**Table 1:** Major clinical features of Sheldon-Hall syndrome.

**Clinical finding**	**Frequency**
*Facial features*	
Triangular face	+++
Small, pointed chin	+++
Micrognatia	+++
Down-slanting palpebral fissures	+++
Prominent nasolabial folds	+++
High arched palate	++
Long philtrum	++
Long face	+
Attached ear lobes	+
Cleft lip/palate	+
*Limbs*	
Ulnar deviation of fingers	+++
Clasped thumb	+++
Overlapping fingers	+++
Hypoplastic and/or absent flexion creases	+++
Camptodactyly	++
Talipes equinovarus	++
Overlapping toes	++
Vertical talus	+
Tarsal fusion	+
*Other features*	
Short stature	+++
Developmental delay	++
Scoliosis	++
Developmental dysplasia of the hip	++
Short neck	+
Webbed neck	+

### Facial manifestations

Among the facial characteristics of SHS the most common finding is a narrow face that is pointed at the chin giving the face a triangular appearance. High arched palate and micrognatia are also very common. Down slanting palpebral fissures are seen in some individuals. Other facial findings include small mouth, high nasal bridge, prominent nasolabial folds, bulbous nose, malar hypoplasia, facial asymmetry, hypertelorism, and posteriorly angulated ears or some other ear abnormalities [4]. Overall facial movement may be diminished.

### Limbs

In an isolated case, the presence of congenital contractures is mandatory in order to entertain the diagnosis of SHS. The joints of the hands, wrists, feet, and ankles are most commonly involved although more proximal joints (*e.g*., elbow, shoulder, knee, and hip) also can be affected. Contractures of the upper limbs typically manifest as overlapping fingers, camptodactyly, and ulnar deviation. Talipes equinovarus, valgus abnormalities, overlapping toes, vertical talus, and tarsal fusion are the most common findings in the lower limbs. The severity of contractures can vary substantially between upper and lower limbs, and the left and right sides of the body. An affected individual might have both equinovarus and calcaneovalgus abnormalities in his/her feet. Contractures are most severe at birth and non-progressive. However, flexion contractures can worsen over time in the absence of appropriate intervention (*e.g*., occupational therapy, physical therapy, surgery).

### Growth and development

Growth parameters during the prenatal and perinatal periods are usually within the range of normal. Gross and fine motor milestones may be achieved somewhat later than age-matched controls but virtually all affected individuals become ambulatory without assist devices. Speech and language are rarely delayed. Cognitive abilities are usually not affected. Life expectancy is normal.

### Other clinical features

Scoliosis, developmental dysplasia of the hips, and short or webbed neck have been reported in some patients [4].

## Etiopathogenesis

SHS is transmitted as an autosomal dominant trait. Familial recurrence has been reported for approximately half of the cases [5].

SHS is caused by mutations in the genes that encode fast twitch skeletal muscle isoforms of troponin I (*TNNI2*) and troponin T (*TNNT3*), and embryonic myosin (*MYH3*) [5-7]. Mutations in these genes explain approximately half of cases studied to date. The remaining cases could be explained by mutations in regulatory regions of *TNNI2*, *TNNT3*, or *MYH3*, large deletions of these genes, or mutations in other as of yet unidentified genes.

The fast twitch skeletal muscle troponin I is encoded by the *TNNI2 *gene which is located on the short arm of chromosome 11 (11p15.5). Troponins are muscle proteins that are part of the contractile apparatus. To date, 4 different *TNNI2 *mutations have been reported in DA2B patients: p.R156X, p.R174Q, p.E167del, and p.K175del [6-10]. All of these mutations have been found in familial cases. p.R156X and p.K175del were each found in 2 families, p.R174Q was reported in 4 families, and p.E167del in a single family. All of the reported mutations map to the C-terminal region of troponin I which is required for the inhibitory action of the protein [6]. The mechanism by which mutations in *TNNI2 *cause contractures is unclear but troponin I binds to actin and tropomyosin, and in the absence of Ca^2+ ^prevents muscle contraction. In vitro and ex vivo contractility studies of troponin I mutants suggest that p.R156X and p.R174Q increase muscle contractility via increasing Ca^2+ ^sensitivity [11] suggesting that these mutations disturb troponin I's ability to regulate Ca^2+ ^levels.

The *TNNT3 *gene, which is near *TNNI2 *on chromosome 11p15.5, was considered a positional and functional candidate [7]. It encodes the fast-twitch skeletal muscle isoform of troponin T and like troponin I forms part of the contractile apparatus of fast twitch myofibers. A missense mutation predicted to cause substitution of an arginine residue with histidine (p.R63H) was found in a mother and her 2 affected children [7]. This mutation maps to a domain of the protein that is not involved in direct interactions with other troponins, but it causes increased ATPase activity and Ca^2+^-dependent force generation [11]. Hence, p.R63H appears to also increase muscle contractility.

After it became evident that SHS was caused by defects of the contractile apparatus of fast-twitch myofibers, we investigated whether SHS could be caused by mutations in genes that encode other proteins of the contractile apparatus [5]. The genes encoding myosin heavy chains, the major component of the contractile unit, were screened in patients in whom no mutation had been found in *TNNI2 *or *TNNT3*, giving priority to genes expressed during prenatal or perinatal development. This eventually led to the screening of the gene that encodes embryonic myosin heavy chain (*MYH3*). Mutations in *MYH3 *were found in 5 of 12 familial (42%) and 7 of 26 sporadic (27%) cases [5]. All but one of these were missense mutations. A p.841del mutation was found in one familial and one sporadic case. Seven missense mutations (p.S261F, p.S292C, p.E375K, p.D517Y, p.G769V, and p.T178I) were located in the region of MYH3 that encodes the head domain of myosin; and two (p.D1622A, p.A1637V) were located in the region that encodes the tail domain. One missense mutation (p.K838E) and the deletion (p.841del) were predicted to disrupt the linker region between the head and the tail domains of MYH3. All of the *MYH3 *mutations were predicted to affect the formation and stability of the contractile apparatus. Collectively, mutations in the *MYH3*, *TNNI2*, and *TNNT3 *genes account for about half of all studied cases of SHS.

## Genotype-phenotype relationship

Extensive within and between family phenotypic variation is observed in SHS. Both the specific joints affected and the degree to which joints are affected varies. The basis of this variability is unclear but could arise by several different mechanisms or a combination thereof as none are mutually exclusive. First, mutation in different genes could cause different phenotypes. However, none of the phenotypic variability between families appears to correspond to disruption of specific proteins much less specific genotypes. In other words, individuals with a mutation in either *TNNI2*, *TNNT3*, or *MYH3 *have phenotypes that cannot be distinguished from one another. Even individuals with the same mutation exhibit widely variable clinical findings. Nevertheless, an important caveat is that a relatively small number of cases with any specific mutation have been studied, and the phenotypic information from many of these cases is incomplete. Overall, more cases need to be studied to rigorously evaluate if there is a substantial correspondence between genotype and phenotype in individuals with SHS.

*MYH3 *mutations that cause SHS are distinct from those that cause FSS [5]. Almost all FSS mutations are predicted to affect a pocket of embryonic myosin which is important in ATP binding and hydrolysis whereas mutations that cause SHS disturb amino acid residues on the surface of embryonic myosin. Therefore, it is reasonable to hypothesize that FSS is caused by defective kinase action, while SHS is caused by the instability of the protein complex.

## Diagnosis

The diagnosis of SHS is based on clinical characteristics [2,4]. The major diagnostic criteria for SHS are ulnar deviation, camptodactyly, overriding fingers, hypoplastic and/or absent flexion creases, talipes equinovarus, calcaneovalgus abnormalities, vertical talus and/or metatarsus varus [2]. Minor diagnostic criteria include a triangular face, downslanting palpebral fissures, attached ear lobules, a small mouth, a small mandible, an arched palate, cervical webbing and short stature. Exclusion criteria include primary neurological and muscle abnormalities. At least 2 of the major diagnostic criteria must be present in at least one member of a family to be classified as affected. In the presence of an individual meeting these criteria inclusion criteria might be relaxed for other family members. In addition, a positive family history can help exclude sporadic disorders with similar features. Because of the phenotypic variability some patients might show atypical presentations. However, congenital contractures of the distal joints, a small mouth, prominent nasolabial folds and a triangular face are consistent findings. The severity of the contractures typically improves with age and the facial characteristics are often less distinctive in adults.

## Antenatal diagnosis

Occasionally women may note decreased fetal movements, but they might also be normal. The clinical findings of SHS can be detected prenatally by ultrasonography. However, detection may not be possible until 18–24 weeks of gestation. If the family history is positive and the mutation is known in the family, prenatal molecular genetic diagnosis is possible [12].

## Genetic counseling

SHS is a monogenic disorder with autosomal dominant inheritance. Affected individuals have a 50% risk of transmitting the disease to their offspring. However, many of the patients reported to date have had *de novo *mutations. The recurrence risk in such families is low but recurrent "*de novo*" mutations in several families with DA suggest that germline mosaicism does occur in DA.

Mutation analysis of the known genes should be offered to affected individuals in certain circumstances (*e.g*., diagnosis is in question). Since the majority of mutations have been found in *MYH3*, this gene should be screened first. If no mutations are found in *MYH3*, *TNNI2 *and *TNNT3 *genes should also be sequenced.

## Differential diagnosis

SHS shares features with DA1 and FSS. DA1 is defined as the prototypic DA, and the clinical findings are limited to the contractures of the distal joints of the hands and feet [13]. Individuals with DA1 do not have major contractures of the facial muscles or facial abnormalities [13].

In SHS the shape of the mouth and chin is different from that of FSS [2,4]. Generally, the facial contractures are not as severe in SHS. Newborns affected with SHS do not have the pinched lips accompanied by the appearance of a "whistling face" nor do they typically have "H" shaped dimpling of the chin. Feeding difficulties at birth and a need for surgical revision of the mouth, which are nearly universal features of children with FSS, are not observed in SHS. The chin is more triangular in individuals affected with SHS. In addition, strabismus and severe scoliosis is less common in SHS than in FSS.

The small mouth may also resemble DA7 (also known as Trismus-Pseudocamptodactyly syndrome), however, SHS does not include trismus of the jaw, which is a unique characteristic of DA7.

## Management

There is no specific treatment for SHS. The contractures occur early in development and they improve postnatally. Although some spontaneous improvement might be expected, prolonged use of braces and casts, or multiple surgeries to correct the limb contractures are often required. Most patients will benefit from physical therapy, which should start soon after birth.

Individuals with SHS have a normal life expectancy. Hearing, vision, speech, and motor development should be checked as appropriate. The majority of the patients will require series of orthodontic treatment as well as prolonged orthopedic management. Surgical procedures can be complicated by difficult intravenous access, laringoscopy, and spinal anesthesia. In addition, there is evidence to suggest that individuals with DA syndromes including SHS are at higher risk for malignant hyperthermia [14].

## Unresolved questions

Almost 50% of the patients affected with SHS, do not have mutations in *MYH3*, *TNNI2*, or *TNNT3 *genes. It needs to be addressed whether these patients have mutations in another gene or mutations in regulatory regions of these genes. With respect to the latter, there are no known families that map to any of the known loci and in whom mutations have not been identified. The molecular mechanism underlying the contractures is not well understood. Some mutations appear to increase contractility, although this has not yet been confirmed in *ex vivo *studies of myofiber contractility in affected individuals. Finally, the SHS phenotype starts to develop *in utero*, suggesting that a clear understanding of how muscle development is perturbed in SHS will require studies in animal models.

## Abbreviations

DA: distal arthrogryposis; FSS: Freeman-Sheldon syndrome; MIM: Mendelian Inheritance in Man; *MYH3*: embryonic myosin heavy chain; SHS: Sheldon-Hall syndrome; *TNNI2*: troponin I2; *TNNT3*: troponin T3.

## Consent

Written consent for publication of the clinical picture was obtained from the patient.

## Competing interests

The authors declare that they have no competing interests.

## Authors' contributions

RMT wrote the first draft of the manuscript; both authors revised the manuscript and approved the final version.
